# Global update on the susceptibility of human influenza viruses to neuraminidase inhibitors, 2014–2015

**DOI:** 10.1016/j.antiviral.2016.06.001

**Published:** 2016-08

**Authors:** Aeron C. Hurt, Terry G. Besselaar, Rod S. Daniels, Burcu Ermetal, Alicia Fry, Larisa Gubareva, Weijuan Huang, Angie Lackenby, Raphael T.C. Lee, Janice Lo, Sebastian Maurer-Stroh, Ha T. Nguyen, Dmitriy Pereyaslov, Helena Rebelo-de-Andrade, Marilda M. Siqueira, Emi Takashita, Masato Tashiro, Danielle Tilmanis, Dayan Wang, Wenqing Zhang, Adam Meijer

**Affiliations:** a WHO Collaborating Centre for Reference and Research on Influenza, VIDRL, Peter Doherty Institute for Infection and Immunity, Melbourne, Victoria 3000, Australia; b Melbourne School of Population and Global Health, University of Melbourne, Parkville, Victoria 3010, Australia; c Global Influenza Programme, World Health Organization, Avenue Appia 20, 1211 Geneva 27, Switzerland; d The Francis Crick Institute, Worldwide Influenza Centre (WIC), Mill Hill Laboratory (formerly the WHO CC for Reference and Research on Influenza, MRC, National Institute for Medical Research), The Ridgeway, London NW7 1AA, UK; e WHO Collaborating Center for the Surveillance, Epidemiology and Control of Influenza, Centers for Diseases Control and Prevention, 1600 Clifton RD NE, MS-G16, Atlanta, GA, United States; f WHO Collaborating Centre for Reference and Research on Influenza, National Institute for Viral Disease Control and Prevention, Collaboration Innovation Center for Diagnosis and Treatment of Infectious Diseases, China CDC, Beijing, China; g National Infection Service, Public Health England, London NW9 5HT, UK; h Bioinformatics Institute, Agency for Science, Technology and Research, 30 Biopolis Street, #07-01, Matrix, Singapore 138671, Singapore; i Public Health Laboratory Centre, 382 Nam Cheong Street, Hong Kong, China; j School of Biological Sciences, Nanyang Technological University, 60 Nanyang Drive, Singapore 637551, Singapore; k National Public Health Laboratory, Ministry of Health, 3 Biopolis Drive, Synapse #05-14 to 16, Singapore 138623, Singapore; l Division of Communicable Diseases, Health Security, & Environment, World Health Organization Regional Office for Europe, UN City, Marmorvej 51, DK-2100 Copenhagen, Denmark; m Instituto Nacional de Saúde, Av. Padre Cruz, 1649-016 Lisboa, Portugal; n Faculdade de Farmácia, Universidade de Lisboa, Av. Prof Gama Pinto, 1649-003 Lisboa, Portugal; o National Influenza Center, Laboratorio de Virus Respiratorios- Oswaldo Cruz Institute/FIOCRUZ-Rio de Janeiro, Brazil; p WHO Collaborating Centre for Reference and Research on Influenza, National Institute of Infectious Diseases, Gakuen 4-7-1, Musashimurayama, Tokyo 208-0011, Japan; q National Institute for Public Health and the Environment, PO Box 1, 3720 BA Bilthoven, The Netherlands

**Keywords:** Influenza virus, Antiviral resistance, Neuraminidase inhibitors, Oseltamivir, Global analysis, Reduced susceptibility

## Abstract

The World Health Organization (WHO) Collaborating Centres for Reference and Research on Influenza (WHO CCs) tested 13,312 viruses collected by WHO recognized National Influenza Centres between May 2014 and May 2015 to determine 50% inhibitory concentration (IC_50_) data for neuraminidase inhibitors (NAIs) oseltamivir, zanamivir, peramivir and laninamivir. Ninety-four per cent of the viruses tested by the WHO CCs were from three WHO regions: Western Pacific, the Americas and Europe. Approximately 0.5% (n = 68) of viruses showed either highly reduced inhibition (HRI) or reduced inhibition (RI) (n = 56) against at least one of the four NAIs.

Of the twelve viruses with HRI, six were A(H1N1)pdm09 viruses, three were A(H3N2) viruses and three were B/Yamagata-lineage viruses. The overall frequency of viruses with RI or HRI by the NAIs was lower than that observed in 2013–14 (1.9%), but similar to the 2012–13 period (0.6%). Based on the current analysis, the NAIs remain an appropriate choice for the treatment and prophylaxis of influenza virus infections.

## Highlights

• A total of 13,312 influenza viruses were collected worldwide, May 2014–May 2015.
• Approximately 0.5% showed reduced inhibition by at least one NA inhibitor.
• The frequency of viruses with reduced inhibition was lower than in 2013–14 (1.9%).
• NA inhibitors remain an appropriate choice for influenza treatment and prophylaxis.
• Global surveillance of influenza antiviral susceptibility should be continued.

## 1 Introduction

The first class of influenza antiviral drugs to be approved, the adamantanes (namely amantadine and rimantadine), continue to be ineffective for the treatment of influenza due to resistance conferred by a S31N amino acid substitution in the M2 protein of virtually all currently circulating A(H1N1)pdm09 and A(H3N2) viruses. The neuraminidase inhibitor (NAI) class of influenza antivirals first came to market in 1999 and now encompasses four compounds – oseltamivir (Tamiflu^®^), zanamivir (Relenza^®^), peramivir (Rapivab^®^) and laninamivir (Inavir^®^) - that differ in their chemical structure, bioavailability and mode of administration. In the majority of countries, only oseltamivir and inhaled zanamivir are approved, with oseltamivir being the most widely used. Together with oseltamivir and zanamivir, peramivir and laninamivir are approved and used in Japan, and peramivir is also approved in China, the Republic of Korea and the USA. The use of influenza antivirals differs around the world; countries such as Japan and the USA use the greatest volumes and regularly treat influenza virus infected patients presenting at general practitioners or hospital outpatient clinics, while other countries primarily use the drugs to treat severely ill hospitalised patients. Aside from the treatment of seasonal influenza, several countries around the world have stockpiled large volumes of influenza antivirals for use in a pandemic situation. Other influenza antivirals that target other viral proteins or host factors, such as nitazoxanide, favipiravir and fludase, are currently in late-phase clinical trials but as yet have not been approved for use in patients with uncomplicated influenza infections. As such there remains a strong reliance on the NAIs, specifically oseltamivir, for the treatment of severely ill patients.

Surveillance for viruses with reduced NAI susceptibility is important to inform pandemic preparedness strategies and ensure that treatment and clinical management guidelines remain appropriate. Here we describe the third global update of NAI susceptibility for viruses collected through the World Health Organization (WHO) Global Influenza Surveillance and Response System (GISRS) for the period May 2014 to May 2015 (subsequently referred to as 2014–15).

Emergence of viruses with reduced NAI susceptibility is not unprecedented and has been observed over the last decade both on a local and global scale. For example, in late 2007 former seasonal A(H1N1) viruses acquired the neuraminidase (NA) H275Y amino acid substitution which conferred oseltamivir resistance, impacted clinical effectiveness (Kawai et al., 2009a, Kawai et al., 2009b), and spread globally in less than 12 months (Dharan et al., 2009, Hurt et al., 2009, Lackenby et al., 2008, Meijer et al., 2009). More recently, clusters of A(H1N1)pdm09 viruses containing NA H275Y substitution have been detected at a local level (Hurt et al., 2011, Takashita et al., 2015a). Two of these clusters, in Hokkaido, Japan and Pennsylvania, USA were described in our last annual report of NAI susceptibility for the 2013–14 period (Takashita et al., 2015b). These events show that some former seasonal A(H1N1) and A(H1N1)pdm09 viruses containing the NA H275Y amino acid substitution are able to replicate and transmit as efficiently as normal wild-type viruses. The presence of other permissive amino acid substitutions are thought to restore the usual deteriorating effect of the NA H275Y substitution on viral fitness (Butler et al., 2014, Abed et al., 2015).

## 2 Overall analysis of phenotypic antiviral susceptibility data from WHO CCs

As part of the WHO GISRS network, over 140 WHO National Influenza Centres (NICs) (http://www.who.int/influenza/gisrs_laboratory/national_influenza_centres/en/) receive and conduct preliminary analyses on influenza viruses collected within their countries. A representative number of these viruses are then forwarded to at least one of five WHO Collaborating Centres (WHO CCs) (Atlanta, USA; Beijing, China; London, United Kingdom; Melbourne, Australia; and Tokyo, Japan) (http://www.who.int/influenza/gisrs_laboratory/collaborating_centres/en/) for more detailed virus characterisation.

Where available, patient-specific data including age, gender, geographic location, healthcare setting, influenza antiviral treatment history and immune status (immunocompromised or immunocompetent) are included in the analysis. Availability of antiviral treatment data was limited for many of the samples tested but for all viruses showing reduced inhibition by any NAI this information was sought retrospectively. Because the majority of viruses analysed were collected from patients at the time of disease onset (i.e. prior to any commencement of antivirals), the data provides insights on the NAI susceptibility of circulating viruses as opposed to the risk of emergence of viruses with reduced susceptibility in NAI treated patients.

Viruses were typically passaged once or twice in MDCK or MDCK-SIAT1 cells by WHO CCs before being analysed by phenotypic NA inhibition assay to assess NAI susceptibility. The NAI assays utilised the fluorescence-based substrate 2’-(4-Methylumbelliferyl)-α-D-N-acetylneuraminic acid, sodium salt hydrate (MUNANA) as previously described (Potier et al., 1979), but with minor modifications in each laboratory (Takashita et al., 2015b). All five WHO CCs tested for susceptibility to oseltamivir and zanamivir, while the Atlanta, Melbourne and Tokyo WHO CCs also tested isolates for susceptibility to peramivir and laninamivir.

As in the two previous global analysis reports (Meijer et al., 2014, Takashita et al., 2015b), to facilitate pooled analysis of the IC_50_ phenotypic data (the concentration of drug required to inhibit standardised amount of NA activity by 50%) generated in the five WHO CCs, data were converted into relative fold-change values based on WHO CC, assay technique and subtype/lineage of virus. Box-and-whisker plots based on log transformed IC_50_ fold-change data were generated using Tukey’s method. Using the standardised definitions for reporting NAI susceptibility data (WHO, 2012), viruses were classified as showing either normal inhibition (NI), reduced inhibition (RI) or highly reduced inhibition (HRI) based on their fold differences compared to the median of viruses from the same type/subtype/lineage showing NI. Briefly, influenza A viruses are classified as RI if they have a 10- to 100-fold higher IC_50_ or HRI if they have a >100-fold higher IC_50_ than the relevant median. For influenza B viruses RI viruses have a 5- to 50-fold higher IC_50_ while HRI viruses have a >50-fold higher IC_50_ than the relevant median.

**Fig. 1:**
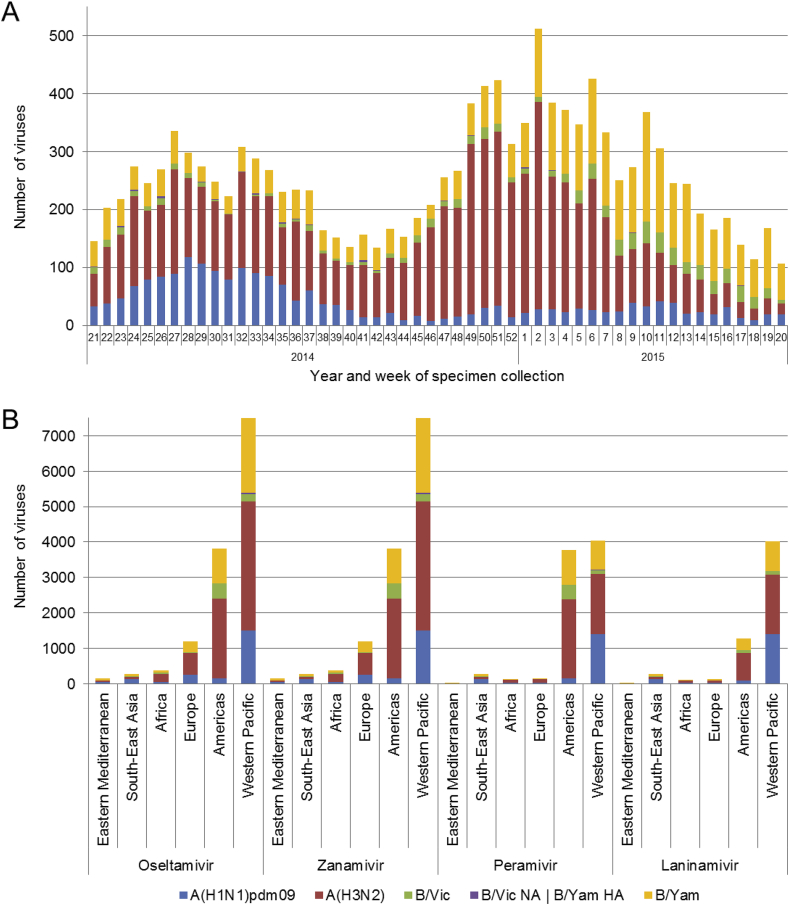
Influenza viruses collected and tested for phenotypic neuraminidase inhibitor (NAI) susceptibility during 2014–2015. A) Week of specimen collection and virus type/subtype/lineage; for specimens tested, peaks in specimen collection during the Southern Hemisphere winter and during the Northern Hemisphere winter were observed. B) Number of viruses tested for phenotypic susceptibility to the four NAIs by World Health Organization region. B/Yamagata-lineage haemagglutinin:B/Victoria-lineage neuraminidase reassortants are shown separately.

Data presented here includes analysis of viruses collected between week 21/2014 (May 19th, 2014) and week 20/2015 (May 17th, 2015). During this period, the number of viruses collected and submitted to WHO CCs peaked during the Southern and Northern Hemisphere winter seasons in weeks 27 (2014) and 2 (2015) respectively (Fig. 1A). Over the 2014–2015 period, a total of 13,312 viruses were tested for NAI susceptibility by the WHO CCs, a 25% increase compared to the number tested in the equivalent 2013–2014 period (Takashita et al., 2015b). The majority of viruses were A(H3N2) (6876; 51.6%) and B/Yamagata-lineage (3594; 27.0%), with lower proportions of A(H1N1)pdm09 (2115; 15.9%) and B/Victoria-lineage viruses (727; 5.5%) (Fig. 1B). In comparison, the predominant influenza virus type/subtype collected and tested in the 2013–2014 period was A(H1N1)pdm09 (48%). As has been observed in previous studies, the majority of viruses tested came from the Western Pacific WHO region (56%), followed by the Americas (29%) and Europe (9%). Only small proportions of viruses were received and tested from the WHO regions of Eastern Mediterranean (1%), South-East Asia (2%) and Africa (3%) (Fig. 1B).

**Fig. 2:**
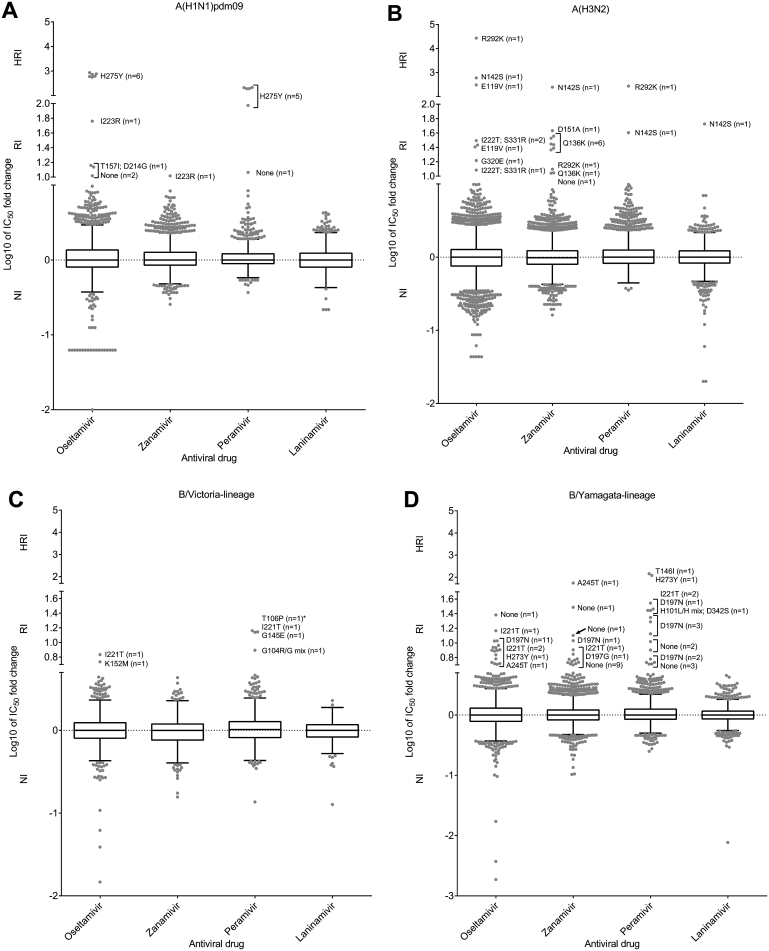
Column-scatter plots of log-transformed 50% inhibitory concentration (IC_50_) fold-change values. Data are presented by virus subtype or lineage (A, A(H1N1)pdm09; B, A(H3N2); C, B/Victoria-lineage; D, B/Yamagata-lineage) and neuraminidase inhibitor (labelled on the X-axis: oseltamivir, zanamivir, peramivir, laninamivir). Panel C also contains B/Yamagata-lineage haemagglutinin:B/Victoria-lineage neuraminidase reassortants, of which the one with amino acid substitution is indicated with an asterisk (*) in the peramivir column. The boxes indicate the 25–75 percentile and the whiskers stretch to the lowest and highest value within 1.5 times the interquartile region value from both the 25 and 75 percentile values respectively (Tukey’s definition). The Y-axes have been split into 3 compartments according to the World Health Organization Antiviral Working Group recommended thresholds for normal inhibition (NI) (A viruses <10-fold; B viruses <5-fold), reduced inhibition (RI) (A viruses 10- to 100-fold; B viruses 5- to 50-fold), and highly reduced inhibition (HRI) (A viruses >100-fold; B viruses >50-fold). For RI and HRI viruses that have been sequenced the determined NA amino acid substitutions are shown; amino acid position numbering is A subtype and B type specific. All viruses were tested for susceptibility to oseltamivir and zanamivir but not all, including some variants, were tested against peramivir and laninamivir.

**Fig. 3:**
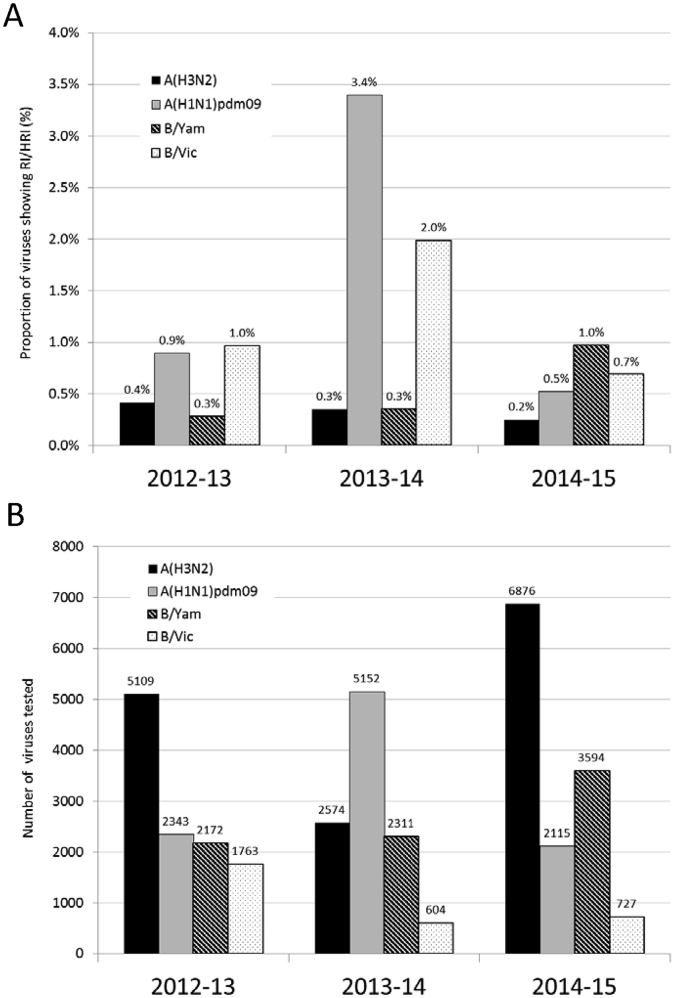
A) Proportions of viruses showing RI or HRI by neuraminidase inhibitors over the 2012–2015 period. B) Number of viruses tested in NA inhibition assays. Data compiled from the global studies reporting on viruses isolated during 2012–13 (Meijer et al., 2014), 2013–14 (Takashita et al., 2015b) and 2014–15 (this study).

Of the 13,312 viruses tested, 68 (0.5%) showed RI or HRI by one or more of the NAIs, a lower proportion than observed during the 2013–14 period (204/10,641; 1.9%) (Fig. 2, Fig. 3; Table 1, Table 2). The NA genes from the 68 RI/HRI viruses were sequenced and 50 were found to contain amino acid substitutions compared to corresponding consensus sequences from wild-type viruses showing NI (Table 1, Table 2). NA genes were sequenced using standard procedures which differed slightly between laboratories. An example protocol from the Melbourne WHO CC is described in Deng et al. (2015) with specific NA segment primers from that procedure listed in Supplementary Table 1. Unfortunately many of the clinical specimens, from which the RI/HRI viruses with NA substitutions were recovered, were unavailable for sequence analysis (n = 30), but of those that were, 18 contained the same NA amino acid substitution present in the isolate, while two did not, suggesting that those substitutions arose during cell culture (Table 1, Table 2).

## 3 A(H1N1)pdm09 viruses showing RI or HRI

Of 2115 A(H1N1)pdm09 viruses tested, 11 (0.5%) showed RI or HRI by one or more of the NAIs (Fig 2; Table 1). Of these, six viruses were NA H275Y variants, showing HRI by oseltamivir (559- to 856-fold greater than the median) and RI/HRI by peramivir (94- to 212-fold greater), while retaining NI by zanamivir and laninamivir (Fig. 2; Table 1). The NA H275Y variant viruses were collected from patients within Australia (n = 3), Hawaii, Ukraine and France. Three of the patients were treated with oseltamivir prior to sample collection, two of whom were immunocompromised (Table 1). Compared with the 2013–2014 period, when 169 NA H275Y variants were detected (3.3% of the number tested) from predominantly Japan, China and USA, there was a substantial reduction in the detection of these variants in the 2014–2015 period, although this may be due to the lower number of A(H1N1)pdm09 viruses circulating.

A single A(H1N1)pdm09 virus with a NA I223R amino acid substitution was isolated from a patient in Bolivia (Fig. 2, Table 1). This virus showed RI by oseltamivir (58-fold greater IC_50_ than the median) and zanamivir (10-fold greater) but retained NI by peramivir and laninamivir (Fig. 2, Table 1). Of the remaining four viruses with RI by one or more of the NAIs, one contained dual T157I/D214G NA amino acid substitutions, while sequence analysis of the other three A(H1N1)pdm09 viruses failed to identify any NA substitutions that were either novel or associated with RI by NAIs (Fig. 2, Table 1).

## 4 A(H3N2) viruses showing RI or HRI

Seventeen (0.2%) out of 6876 A(H3N2) viruses showed RI or HRI by one or more of the NAIs. Seven of these contained NA Q136K amino acid substitution that confers RI by zanamivir (Fig. 2, Table 1). This substitution has typically been reported to arise during cell culture propagation (Little et al., 2015) but unfortunately, for the seven isolates with this substitution, none of the respective clinical specimens were available to determine if this was the case. Three A(H3N2) viruses contained dual I222T/S331R amino acid substitutions that resulted in RI by oseltamivir (Fig. 2, Table 1). As single substitutions, neither I222T nor S331R alone confers sufficient IC_50_ rises to be classified as RI, but as a combination they synergistically raised oseltamivir IC_50_ by 12- to 31-fold (Table 1).

Five viruses with RI/HRI due to various NA amino acid substitutions were isolated from patients undergoing NAI treatment (Table 1). Two patients shed viruses containing NA E119V amino acid substitution that conferred RI/HRI by oseltamivir (25- to 303-fold) (Fig. 2), one received oseltamivir while the other (from Japan) received oseltamivir and peramivir and was immunocompromised (Table 1). Another immunocompromised patient from Japan, who was infected with A(H3N2) influenza and treated with oseltamivir and peramivir, shed viruses containing NA R292K amino acid substitution which conferred a massive 27,416-fold increase in oseltamivir IC_50_ as well as conferring HRI by peramivir (271-fold) and RI by zanamivir (12-fold) (Fig. 2; Table 1).

A NA D151A amino acid substitution was detected in an isolate that had RI by zanamivir (43-fold), but sequencing showed that the substitution was not present in the clinical specimen, thereby suggesting that it had arisen during cell culture, as has been reported previously for this substitution (Okomo-Adhiambo et al., 2010). Two other A(H3N2) viruses with less commonly identified NA amino acid substitutions were also detected in 2014–15. The first was a virus from the USA that contained NA N142S amino acid substitution that unusually conferred HRI or RI by all four NAIs (HRI to oseltamivir and zanamivir and RI to peramivir and laninamivir), while the second virus contained NA G320E amino acid substitution that caused RI by oseltamivir (17-fold) (Fig. 2; Table 1).

## 5 B/Victoria-lineage viruses showing RI or HRI

Five (0.7%) of the 727 B/Victoria-lineage viruses (which included 35 B/Yamagata-lineage HA:B/Victoria-lineage NA reassortants) showed RI or HRI by one or more of the NAIs. Three viruses exhibited RI by peramivir and had IC_50_ values that were 8- to 14-fold higher than the median, and contained single NA amino acid substitutions of T106P, G104R/G and G145E respectively (Fig. 2; Table 2). A virus from Honduras with NA I221T amino acid substitution showed RI by both oseltamivir (7-fold) and peramivir (14-fold) and a virus from Bangladesh contained NA K152M amino acid substitution that resulted in a 5-fold increase in oseltamivir IC_50_ (Fig. 2; Table 2). Unfortunately, no information about antiviral treatment or the immune status of the patients was available for these five cases.

## 6 B/Yamagata-lineage viruses showing RI or HRI

Of the 3594 B/Yamagata-lineage viruses tested, 35 (1.0%) had RI or HRI by one or more of the NAIs. Twelve of these viruses contained NA D197N amino acid substitution that conferred 4- to 11-fold increases in oseltamivir IC_50_, 2- to 11-fold increases in zanamivir IC_50_ and 5- to 29-fold increases in peramivir IC_50_ (Fig. 2; Table 2). Three NA D197N variants were from Australia, three from the USA, five from China and one from the Ukraine (Table 2). Interestingly, the three viruses from Australia had identical HA and NA sequences and two of these (B/South Australia/2/2015 and B/South Australia/5/2015) were from a father and son who lived together. While there was no epidemiological link to the third Australian case, it is noteworthy that the specimen collection date differed by only 7 days from that which yielded B/South Australia/2/2015 virus. One of the NA D197N variants from China (B/Tianjin-Hedong/834/2015) was collected in the same time period as the Australian viruses and the HA and NA sequences of these four viruses clustered closely in the phylogenies (Supplementary Fig. S1). Two Chinese NA D197N variants, collected in September and October 2014 (B/Anhui-Baohe/1680/2014 and B/Shanghai-Pudongxin/11568/2014), also had identical HA and NA sequences. From phylogenetic analyses of these variants over the last five years, we also identified an early 2014 cluster of six Japanese viruses with NA D197N substitution that had identical HA and NA sequences, further suggesting that these viruses can spread in the community (Supplementary Fig. S1). For NA D197N variants where the respective clinical specimens were available (n = 4), all contained the amino acid substitution and none of the patients had been treated with an NAI. Three influenza B NA D197N variants (2 from the B/Yamagata-lineage and one from the B/Victoria-lineage) were detected through phenotypic analysis in the equivalent 2013–2014 period, one of which was collected from a patient being treated with zanamivir (Takashita et al., 2015b). Another B/Yamagata-lineage virus from Spain carried NA D197G amino acid substitution which conferred a 5-fold increase in oseltamivir IC_50_; unfortunately the clinical specimen yielding this virus isolate was not available (Fig. 2; Table 2).

Three viruses with NA I221T amino acid substitution that conferred RI by oseltamivir (8- to 15-fold), zanamivir (4- to 5-fold) and peramivir (28- to 35-fold) were collected from patients in Japan, Russia and China (Fig. 2; Table 2). Three other B/Yamagata-lineage viruses, all with different NA amino acid substitutions (T146I, H273Y and A245T), demonstrated HRI by either peramivir or zanamivir (Fig. 2; Table 2), but sequence analysis of the relevant clinical specimen revealed that the T146I substitution arose during cell culture. A further 15 B/Yamagata-lineage viruses showed RI by at least one of the NAIs but sequence analysis failed to reveal any unique NA amino acid substitutions that were considered to be associated with the change in IC_50_ (Fig. 2; Table 2).

## 7 Frequency of RI and HRI conferring NA amino acid substitutions in sequence databases

The interrogation of public sequence databases to determine the frequency of NA amino acid substitutions that confer RI or HRI by the NAIs is a useful adjunct to the phenotypic data generated by the WHO CCs. Analysis of the Global Initiative on Sharing All Influenza Data (GISAID) (www.gisaid.org) and National Center for Biotechnology Information – Influenza Virus Resource (NCBI-IVR) (www.ncbi.nlm.nih.gov/genomes/FLU/FLU.html) databases showed that sequences from 7316 influenza viruses collected during the 2014–15 period had been deposited, of which 2440 (33%) were from viruses that had not been tested by the WHO CCs in the phenotypic analysis described above.

We analysed the NA sequences from the 2440 viruses (466 A(H1N1)pdm09; 1373 A(H3N2); 58 B/Victoria-lineage and 543 B/Yamagata-lineage) to determine the number that contained amino acid substitutions associated with reduced inhibition, as listed in the summary table provided by the AVWG on the WHO website (http://www.who.int/influenza/gisrs_laboratory/antiviral_susceptibility/avwg2014_nai_substitution_table.pdf). Of the 466 A(H1N1)pdm09 virus NA sequences, four contained the H275Y amino acid substitution (from France (n = 2), Norway (n = 1) and Spain (n = 1) that confers HRI by oseltamivir and peramivir, while the E119V amino acid substitution, which confers RI/HRI by oseltamivir, was present in one of the 1373 A(H3N2) NA sequences. Four NA sequences from the 543 B/Yamagata-lineage viruses contained the same D197N amino acid substitution that was detected in twelve variants tested in the phenotypic assay, and which conferred RI by oseltamivir, zanamivir and peramivir; in addition one NA sequence contained the H273Y amino acid substitution which confers HRI by peramivir.

## 8 Concluding remarks

Based on our current analysis, >99% of influenza viruses tested during 2014–15 were susceptible to all four NAIs, indicating that these antivirals remain an appropriate choice for the treatment and prophylaxis of influenza virus infections. Viruses with RI or HRI occurred across multiple regions and were not detected at higher frequencies in countries where NAI usage is greatest (i.e. Japan and the USA) (Supplementary Table 2). This is the third global update on influenza NAI susceptibility based on data generated by the WHO CCs’ analyses of viruses received from NICs throughout the world. The overall frequency of viruses with RI or HRI by the NAIs was highest in 2013–14 (1.9%), compared to 2012–13 and the most recent 2014–15 period where the frequencies were 0.6% and 0.5% respectively (Fig. 3). The higher frequency of RI/HRI viruses in 2013–14 was due mainly to A(H1N1)pdm09 being the dominant influenza A subtype with an associated increased detection of A(H1N1)pdm09 viruses with NA H275Y amino acid substitution, with clusters of cases being detected in Japan, the USA and China (Takashita et al., 2015b). Of the different types/subtypes, B/Yamagata-lineage viruses had the highest frequency of RI/HRI viruses in the 2014–15 period, of which variants with NA D197N amino acid substitution were the most prevalent. Given the close genetic similarity across the HA and NA of some NA D197N variants in Australia and China it is possible that some low level community transmission of these viruses had occurred, so close monitoring of influenza B viruses for this substitution will be important in upcoming influenza seasons.

## Contributions

All WHO-AVWG members, WHO Headquarters and Regional Office Staff present during the 5th WHO-AVWG meeting held 25–26 June 2015 in Geneva were involved in discussions relating to this global update. AH drafted the manuscript and all authors contributed to editing the final manuscript. AH, DT, BE, RD, LG, HTN, ET, MT, DW and WH generated and provided the NAI sensitivity data and molecular analysis. AM performed analysis of the data from the WHO CCs, RL and SM-S performed the analysis of data from sequence databases and RD and BE generated the phylogenies presented in supplemental data.

## Disclaimer

The authors alone are responsible for the views expressed in this article and they do not necessarily represent the views, decisions or policies of the institutions with which they are affiliated.

## Figures and Tables

**Table 1:** Virus and patient characteristics of 28 influenza A viruses showing RI or HRI, tested by WHO CCs.^a^

Virus	n	IC_50_ fold-change compared to reference median IC_50_ values^b^				NA-substitution^c^		Patient setting	Antiviral treatment prior to specimen collection
		Oseltamivir	Zanamivir	Peramivir	Laninamivir	Virus isolate	Clinical specimen		
A(H1N1)pdm09; N = 2115	6	**559–856**	0.9–1.9	**94 – 212 (5)**	1.5–2.6 (5)	H275Y	H275Y (4) Not available (2)	GP/Outpatient (1) Hospital (3) Unknown (2)	Yes, oseltamivir (2) Yes, antiviral unknown (1) No (2) Unknown (1)
	1	**14**	7.0	1.6	1.3	T157I, D214G	No sequence available, poor quality data	GP/Outpatient	No
	1	**58**	**10**	7.2	2.2	I223R	I223R	Unknown	Unknown
	2	**10–14**	2.2–8.4	3.7 (1)	3.6 (1)	None	Not available (1) Not sequenced (1)	GP/Outpatient	No (1) Unknown (1)
	1	1.6	2.0	**12**	1.9	None	Not sequenced	Hospital	No
A(H3N2); N = 6876	7	0.3–1.1	**11–36**	2.9–9.0 (2)	2.0–3.6 (2)	Q136K	Not available	Hospital (5) Unknown (2)	Unknown
	3	**12–31**	2.9–7.2	1.6–2.4	2.5–3.6	I222T, S331R	I222T, S331R	GP/Outpatient	No
	2	**25–303**	1.5–2.3	1.5–2.1	1.1–2.2	E119V	E119V	Hospital (1) Unknown (1)	Yes, oseltamivir (1), oseltamivir and peramivir (1)
	1	**595**	**244**	**40**	**53**	N142S	N142S	Unknown	Unknown
	1	2.9	**43**	9.9	6.9	D151A	Substitution not present	GP/Outpatient	Yes, zanamivir
	1	**27 146**	**12**	**271**	2.7	R292K	R292K	Hospital	Yes, oseltamivir and peramivir
	1	**17**	8.4	3.3	1.9	G320E	Not available	Unknown	Unknown
	1	7.9	**11**	nd	nd	None	Not available	GP/Outpatient	Yes, oseltamivir

a Between brackets the number of viruses for which data was reported if less than the number reported in column ‘n’. RI = reduced inhibition; HRI = highly reduced inhibition; nd = not done; None = no amino acid substitutions compared to viruses with NI phenotype.

b RI and HRI fold-change values are displayed underlined and in bold typeface.

c Amino acid position numbering is A subtype specific.

**Table 2:** Virus and patient characteristics of 40 influenza B viruses showing RI or HRI, tested by WHO CCs.^a^

Virus	n	IC_50_ fold-change compared to reference median IC_50_ values^b^				NA-substitution^c^		Patient setting	Antiviral treatment prior to specimen collection
		Oseltamivir	Zanamivir	Peramivir	Laninamivir	Virus isolate	Clinical specimen		
B/Victoria- lineage; N = 727^d^	1	1.1	2.9	**14**	0.7	T106P^d^	Not available	Unknown	Unknown
	1	4.2	0.8	**7.8**	0.7	G104R/G mix	Not available	Unknown	Unknown
	1	0.7	0.7	**14**	0.4	G145E	Not available	Unknown	Unknown
	1	**5.4**	0.6	1.9	1.1	K152M	Not available	Unknown	Unknown
	1	**6.8**	3.7	**14**	1.4	I221T	Not available	Unknown	Unknown
B/Yamagata- lineage; N = 3594	12	**4.4–11**	**2.2–11**	**5.4 – 29 (6)**	1.6–3.3 (6)	D197N	D197N (4) Not available (7) Not sequenced (1)	GP/Outpatient (4) Hospital (5) Unknown (2)	No (3) Unknown (8)
	3	**7.9–15**	**4.1–5.4**	**28–35**	1.7–1.9	I221T	I221T (1) Not available (2)	GP/Outpatient (1) Hospital (1) Unknown (1)	No (1) Unknown (2)
	1	1.7	1.8	**28**	1.2	H101L/H mix, D342S	Not available	Unknown	Unknown
	1	2.0	1.0	**145**	0.8	T146I	Substitution not present	GP/Outpatient	No
	1	3.3	**5.4**	nd	nd	D197G	Substitution not present	GP/Outpatient	Yes, oseltamivir
	1	**5.2**	1.0	**120**	0.6	H273Y	H273Y	Unknown	Unknown
	1	**8.1**	**55**	nd	nd	A245T	Not available	Hospital	Unknown
	15	**0.8–24**	**0.2–31**	**0.8 – 10 (14)**	1.3–4.2 (14)	None	Not available	GP/Outpatient (7) Hospital (1) Unknown (7)	No (1) Unknown (14)

a Between brackets the number of viruses for which data was reported if less than the number reported in column ‘n’. RI = reduced inhibition; HRI = highly reduced inhibition; nd = not done; None = no amino acid substitutions compared to viruses with NI phenotype.

b RI and HRI fold-change values are displayed underlined and in bold typeface.

c Amino acid position numbering is B type specific.

d 35 of these viruses are B/Yamagata-lineage haemagglutinin (HA) – B/Victoria-lineage neuraminidase (NA) reassortants; because the NA defines the IC_50_ values these viruses are listed in the B/Victoria-lineage category. One of these viruses had NA T106P.

## Appendix A Supplementary data

Supplementary Fig. S1Phylogenetic tree of HA sequences (A) and NA sequences (B), identifying variant influenza B viruses with a D197N amino acid substitution in red. Reference viruses are coloured in black, viruses collected in different years are coloured: 2015 pink, 2014 green and 2013 brown.
