# Supplementary material for: Global update on the susceptibility of human influenza viruses to neuraminidase inhibitors, 2014–2015
Source: Antiviral Res. 2016 Aug;132:178–85. doi: 10.1016/j.antiviral.2016.06.001 (PMC5357725; doi:10.1016/j.antiviral.2016.06.001)
Supplement: Supplementary file 3 [file mmc3.pdf]

Resistant viruses
Reference viruses

Collection date
2015
2014
2013
