# Supplementary material for: Global update on the susceptibility of human influenza viruses to neuraminidase inhibitors, 2014–2015
Source: Antiviral Res. 2016 Aug;132:178–85. doi: 10.1016/j.antiviral.2016.06.001 (PMC5357725; doi:10.1016/j.antiviral.2016.06.001)
Supplement: Supplementary file 4 [file mmc4.docx]

Supplementary Table 1. Primers used for the RT-PCR and sequencing of NA gene segments

| **RT-PCR primers** | |  |
| --- | --- | --- |
|  |  |  |
| A(H1N1)pdm09 NA segment |  |  |
| Fragment 1 | pdmNAIMI3F | TGTAAAACGACGGCCAGTAGCAAAAGCAGGAG |
|  | pdmNAIMI3R | CAGGAAACAGCTATGACCGTGATAATTAGGGGCATTC |
| Fragment 2 | pdmNAIIMI3F | TGTAAAACGACGGCCAGTGACAGGCCTCATACAAGATCTTC |
|  | pdmNAIIMI3R | CAGGAAACAGCTATGACCAATTACTTGTCAATGG |
|  |  |  |
| A(H3N2)  NA segment |  |  |
| Fragment 1 | NU 1-M13F | TGTAAAACGACGGCCAGTAGCAAAAGCAGGAGT |
|  | N2 1-M13R | CAGGAAACAGCTATGACCCGACATGCTGAGCACTYCCTGAC |
| Fragment 2 | N2 2-M13F | TGTAAAACGACGGCCAGTGAACTTGTRCAGTRGTAATG |
|  | NU 2-M13R | CAGGAAACAGCTATGACCAGTAGAAACAAGGAG |
|  |  |  |
| B  NA segment |  |  |
| Fragment 1 | BNA 1-M13F | TGTAAAACGACGGCCAGTAGCAGAAGCAGAGCATCTTC |
|  | BNA 1-M13R | CAGGAAACAGCTATGACCGATAACAATYTCCYCCGATGC |
| Fragment 2 | BNA 2-M13F | TGTAAAACGACGGCCAGTAAGAAAGTGCYTGYAATTGCA |
|  | BNA 2-M13R | CAGGAAACAGCTATGACCAGTAGTAACAAGAGCAT |
|  |  |  |
| **Sequencing primers** | |  |
|  |  |  |
|  | M13F | TGTAAAACGACGGCCAGT |
|  | M13R | CAGGAAACAGCTATGACC |
