# Supplementary material for: Global update on the susceptibility of human influenza viruses to neuraminidase inhibitors, 2014–2015
Source: Antiviral Res. 2016 Aug;132:178–85. doi: 10.1016/j.antiviral.2016.06.001 (PMC5357725; doi:10.1016/j.antiviral.2016.06.001)
Supplement: Supplementary file 5 [file mmc5.docx]

Supplementary Table 2. Location, week and year of collection of viruses with reduced or highly reduced inhibition.

| Year | Week | Home country patient | Type | A subtype/B lineage | Oseltamivir^1^ | Zanamivir^1^ | Peramivir^1^ | Laninamivir^1^ | Substitution in isolate |
| --- | --- | --- | --- | --- | --- | --- | --- | --- | --- |
| 2014 | 21 | Malaysia | B | B Yam | NI | NI | RI | NI | H101L/H, D342S |
| 2014 | 21 | Macau, China | A | A(H3N2) | NI | RI | NI | NI | Q136K |
| 2014 | 22 | Honduras | B | B Vic | RI | NI | RI | NI | I221T |
| 2014 | 23 | Australia | A | A(H1N1)pdm09 | HRI | NI | HRI | NI | H275Y |
| 2014 | 24 | France | A | A(H1N1)pdm09 | HRI | NI | Not tested | Not tested | H275Y |
| 2014 | 25 | Bangladesh | B | B Vic | RI | NI | NI | NI | K152M |
| 2014 | 25 | Australia | A | A(H1N1)pdm09 | RI | NI | NI | NI | None detected |
| 2014 | 25 | New Zealand | B | B Yam | RI | RI | RI | NI | None detected |
| 2014 | 26 | Australia | A | A(H1N1)pdm09 | RI | NI | NI | NI | T157I , D214G |
| 2014 | 27 | China | B | B Yam | RI | NI | Not tested | Not tested | D197N |
| 2014 | 27 | New Zealand | A | A(H1N1)pdm09 | NI | NI | RI | NI | None detected |
| 2014 | 28 | Bolivia | A | A(H1N1)pdm09 | RI | RI | NI | NI | I223R |
| 2014 | 28 | Lao People's Democratic Republic | B | B Yam | NI | NI | RI | NI | None |
| 2014 | 33 | Lao People's Democratic Republic | B | B Yam | NI | NI | RI | NI | None |
| 2014 | 34 | Lao People's Democratic Republic | B | B Yam | NI | NI | RI | NI | None |
| 2014 | 34 | Australia | A | A(H3N2) | NI | RI | NI | NI | Q136K |
| 2014 | 35 | China | B | B Yam | RI | NI | Not tested | Not tested | I221T |
| 2014 | 36 | China | B | B Yam | RI | NI | Not tested | Not tested | D197N |
| 2014 | 36 | Bangladesh | B | B Vic | NI | NI | RI | NI | G104G/R |
| 2014 | 36 | Lao People's Democratic Republic | B | B Yam | NI | NI | RI | NI | None |
| 2014 | 37 | China | B | B Yam | RI | NI | Not tested | Not tested | D197N |
| 2014 | 39 | Australia | A | A(H1N1)pdm09 | HRI | NI | HRI | NI | H275Y |
| 2014 | 39 | Australia | A | A(H1N1)pdm09 | HRI | NI | HRI | NI | H275Y |
| 2014 | 41 | China | B | B Yam | RI | NI | Not tested | Not tested | D197N |
| 2014 | 41 | Hawaii | A | A(H1N1)pdm09 | HRI | NI | HRI | NI | H275Y |
| 2014 | 41 | China | B | B (Yam HA/Vic NA) | NI | NI | RI | NI | T106P |
| 2014 | 42 | California | A | A(H3N2) | HRI | HRI | RI | RI | N142S |
| 2014 | 44 | China | A | A(H3N2) | NI | RI | Not tested | Not tested | Q136K |
| 2014 | 45 | Mexico | B | B Vic | NI | NI | RI | NI | G145E |
| 2014 | 46 | China | A | A(H3N2) | NI | RI | Not tested | Not tested | Q136K |
| 2014 | 47 | Washington | A | A(H3N2) | HRI | NI | NI | NI | E119V |
| 2014 | 49 | Japan | A | A(H3N2) | NI | RI | NI | NI | D151A |
| 2014 | 49 | China | A | A(H3N2) | NI | RI | Not tested | Not tested | Q136K |
| 2014 | 51 | Japan | A | A(H3N2) | RI | NI | NI | NI | I222T, S331R |
| 2014 | 51 | China | A | A(H3N2) | NI | RI | Not tested | Not tested | Q136K |
| 2014 | 51 | Luxembourg | A | A(H1N1)pdm09 | RI | NI | Not tested | Not tested | None detected |
| 2014 | 52 | China | A | A(H3N2) | NI | RI | Not tested | Not tested | Q136K |
| 2015 | 2 | Lao People's Democratic Republic | B | B Yam | NI | RI | NI | NI | None |
| 2015 | 2 | Australia | B | B Yam | NI | NI | HRI | NI | T146I |
| 2015 | 3 | Japan | A | A(H3N2) | RI | NI | NI | NI | I222T, S331R |
| 2015 | 3 | Japan | A | A(H3N2) | RI | NI | NI | NI | I222T, S331R |
| 2015 | 4 | China | B | B Yam | RI | HRI | Not tested | Not tested | A245T |
| 2015 | 4 | Russian Federation | A | A(H3N2) | RI | NI | NI | NI | G320E |
| 2015 | 4 | Spain | A | A(H3N2) | NI | RI | Not tested | Not tested | None detected |
| 2015 | 5 | Colorado | B | B Yam | RI | NI | RI | NI | D197N |
| 2015 | 5 | Ukraine | B | B Yam | RI | RI | Not tested | Not tested | D197N |
| 2015 | 5 | Japan | A | A(H3N2) | RI | NI | NI | NI | E119V |
| 2015 | 6 | Spain | B | B Yam | NI | RI | Not tested | Not tested | D197G |
| 2015 | 6 | Australia | B | B Yam | RI | NI | RI | NI | D197N |
| 2015 | 6 | China | B | B Yam | RI | NI | Not tested | Not tested | D197N |
| 2015 | 7 | Australia | B | B Yam | RI | NI | RI | NI | D197N |
| 2015 | 7 | Russian Federation | B | B Yam | RI | NI | RI | NI | I221T |
| 2015 | 8 | Japan | B | B Yam | NI | RI | NI | NI | None |
| 2015 | 9 | Australia | B | B Yam | RI | NI | RI | NI | D197N |
| 2015 | 9 | Japan | B | B Yam | NI | RI | NI | NI | None |
| 2015 | 10 | Ukraine | A | A(H1N1)pdm09 | HRI | NI | HRI | NI | H275Y |
| 2015 | 11 | Japan | A | A(H3N2) | HRI | RI | HRI | NI | R292K |
| 2015 | 13 | California | B | B Yam | RI | NI | HRI | NI | H273Y |
| 2015 | 13 | Japan | B | B Yam | RI | RI | RI | NI | I221T |
| 2015 | 13 | Ghana | B | B Yam | NI | RI | Not tested | Not tested | None detected |
| 2015 | 14 | Vermont | B | B Yam | RI | NI | RI | NI | D197N |
| 2015 | 14 | Japan | B | B Yam | NI | RI | NI | NI | None |
| 2015 | 16 | Japan | B | B Yam | NI | RI | NI | NI | None |
| 2015 | 16 | Japan | B | B Yam | NI | RI | NI | NI | None |
| 2015 | 18 | Japan | B | B Yam | NI | RI | NI | NI | None |
| 2015 | 20 | Florida | B | B Yam | NI | NI | RI | NI | D197N |
| 2015 | 20 | Lao People's Democratic Republic | B | B Yam | NI | RI | NI | NI | None |
| 2015 | 20 | Taiwan | B | B Yam | NI | RI | NI | NI | None |

^1^ NI = normal inhibition; RI = reduced inhibition; HRI = highly reduced inhibition;
